# Insecticidal Activity of Bacteria from Larvae Breeding Site with Natural Larvae Mortality: Screening of Separated Supernatant and Pellet Fractions

**DOI:** 10.3390/pathogens9060486

**Published:** 2020-06-18

**Authors:** Handi Dahmana, Didier Raoult, Florence Fenollar, Oleg Mediannikov

**Affiliations:** 1 IRD, AP-HM, MEPHI, Aix Marseille University, 13005 Marseille, France; handi.dahmana@etu.univ-amu.fr (H.D.); didier.raoult@gmail.com (D.R.); 2 IHU-Méditerranée Infection, 13005 Marseille, France; florence.fenollar@univ-amu.fr; 3 IRD, AP-HM, SSA, VITROME, Aix Marseille University, 13005 Marseille, France

**Keywords:** mosquito-borne disease, bacteria, biological control, insecticidal activity, bacteria secondary metabolites, Bacillus

## Abstract

Mosquitoes can transmit to humans devastating and deadly pathogens. As many chemical insecticides are banned due to environmental side effects or are of reduced efficacy due to resistance, biological control, including the use of bacterial strains with insecticidal activity, is of increasing interest and importance. The urgent actual need relies on the discovery of new compounds, preferably of a biological nature. Here, we explored the phenomenon of natural larvae mortality in larval breeding sites to identify potential novel compounds that may be used in biological control. From there, we isolated 14 bacterial strains of the phylum Firmicutes, most of the order Bacillales. Cultures were carried out under controlled conditions and were separated on supernatant and pellet fractions. The two fractions and a 1:1 mixture of the two fractions were tested on L3 and early L4 *Aedes albopictus*. Two concentrations were tested (2 and 6 mg/L). Larvae mortality was recorded at 24, 48 and 72 h and compared to that induced by the commercialized *B. thuringiensis* subsp. *israelensis*. Of the 14 strains isolated, 11 were active against the *A. albopictus* larvae: 10 of the supernatant fractions and one pellet fraction, and mortality increased with the concentration. For the insecticide activity prediction in three strains of the *Bacillus cereus* complex, PCR screening of the crystal *(Cry)* and cytolytic *(Cyt)* protein families characteristic to *B. thuringiensis* subsp. *israelensis* was performed. Most of the genes coding for these proteins’ synthesis were not detected. We identified bacterial strains that exhibit higher insecticidal activity compared with a commercial product. Further studies are needed for the characterization of active compounds.

## 1. Introduction 

Diseases transmitted by arthropods participate in a major part to infectious diseases’ morbidity and mortality worldwide, and mosquitoes are number one in terms of public health importance, transmitting such deadly diseases as malaria (*Anopheles*), filariasis (*Culex*) and dengue (*Aedes*) that cause millions of deaths every year [1]. To control them, a large number of chemical compounds have been used. However, their intensive use resulted in numerous drawbacks such as the contamination of water and food sources, poisoning of non-target fauna and flora, concentration in the food chain and resistance [2]. The use of biological control and biopesticides based on natural products appears to be the best alternative. 

The interest in the effectiveness of microorganisms and their metabolites for insect control dates back decades. Approximately 1500 naturally occurring microorganisms have been identified as potentially insecticidal agents [1] and metabolites from 942 microbial isolates were screened for insecticidal properties [3]. *Bacillus* spp. are ubiquitous bacteria found in a wide range of terrestrial and aquatic environments and are known for their wide range of physiological abilities allowing them to grow in every environment and to compete desirably with other organisms, especially through their ability to form extremely resistant spores and to produce metabolites [4]. Prior to the discovery of *Bacillus thuringiensis* subsp. *israelensis* (*Bti*) and *Lysinibacillus sphaericus*, bacteria had not received much attention as a source of promising molecules for the control of insects of medical and veterinary importance. Nonetheless, *Bti* and *L. sphaericus* products now dominate the commercial market for the control of mosquito larvae [5]. 

Among the different classes of proteins produced by *B. thuringiensis*, three classes of larvicidal proteins are well-known: *Cry* (crystal proteins), *Cyt* (cytolytic toxicity) and *Vip* (proteins throughout the vegetative phase), some of which are toxic against a wide range of insect orders, nematodes and human cancer cells. It was demonstrated that *Cyt* toxins possess less toxicity against mosquito larvae than *Cry* toxins. Numerous factors may increase the risk of acquiring resistance in target insects and also have a negative impact on non-target insects [6,7,8,9]. Several proteins are toxic for dipteran insects such as *Cry2*, *Cry4*, *Cry10*, *Cry11*, *Cry16*, Cry17, *Cry19* and *Cyt*, and several general and specific sets of primers we designed in order to screen *Bacillus* spp. isolates for their presence [10]. 

The selection of resistance to *Bti* in mosquitoes has been achieved under laboratory conditions [11] but despite having been used for several decades, there is little evidence for the selection of *Bti* resistance under field conditions [9]. In contrast, various levels of laboratory and field resistance are widely found in *L. sphaericus* products, depending on prior exposure to naturally existing strains, population genetic background and gene exchange with untreated populations, as well as product application strategies [12]. 

Several studies have been carried out by isolating large collections of *Bacillus* strains to explore their biological activities, including antibacterial, insecticide and others [1,3,13,14,15,16]. Most of these beneficial compounds are largely used in several medical or economic purposes, especially to control various diseases in animals, humans and plants, when applied as a biological control agent [13]. Different studies investigated the bacterial populations of breeding sites and their possible effects on mosquito larval populations [17]. New *Bacillus* isolates were reported harboring new δ-endotoxin genes and/or different forms of the known endotoxin genes and considered very promising in the search of novel endotoxins [16]

In the present study, we proceeded as follows:

- We explored 20 larval breeding sites that have never been treated with insecticides, looking for possible mosquito larval mortality. Only one had a natural larva mortality;
- We isolated bacterial strains from this larva breeding site that presented a natural mortality of mosquito larvae;
- We tested the bacteria-free supernatant and disrupted bacterial pellet separately, or the mixture of both of the different strains of bacteria.

We suspected that bacterial insecticide may explain this phenomenon and attempted to explore it.

## 2. Results 

### 2.1. Isolated Strains 

A total of 173 strains from 9 different genera were isolated. Since *Bacillales* are known for their entomopathogenic activity, we focused our study on strains belonging to the Firmicutes phylum, corresponding to 14 of the isolated strains that have been deposited in the CSUR collection of IHU Mediterranée Infection. (Table 1).

**Figure 1:**
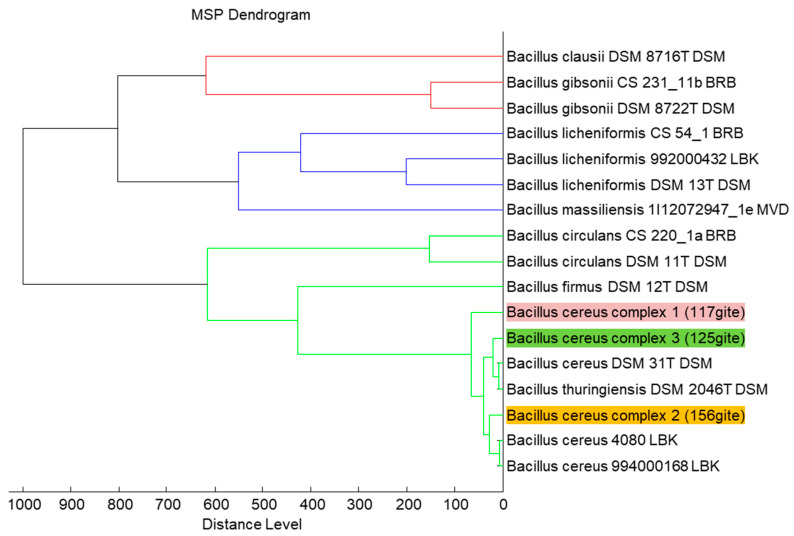
Dendrogram of *Bacillus* spp. strains representing the three clusters of *B. cereus* complex generated using the MALDI Biotyper 3.0 software (Bruker Daltonics, Bremen, Germany) with other *Bacillus* spp. as the outgroup.

**Figure 2:**
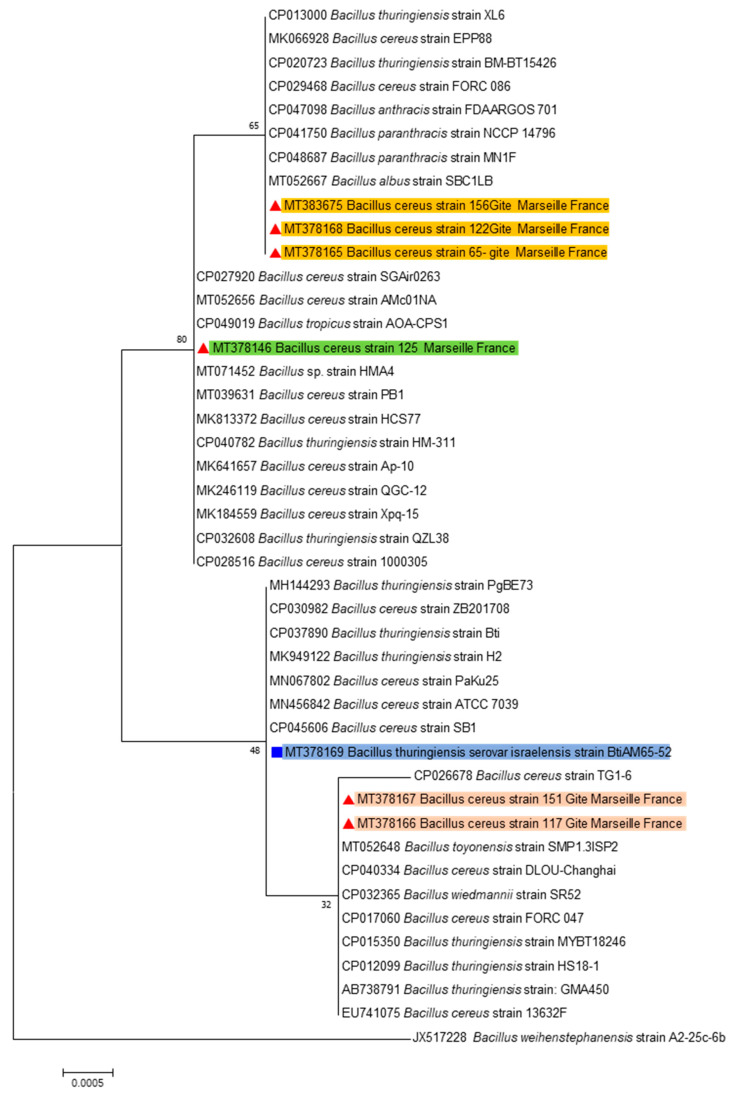
Maximum-likelihood phylogenetic tree of *Bacillus* spp., representing the three clusters of the *B. cereus* complex and including the *Bti* AM65-52 based on long 16S RNA gene (1500-bp).

After identification by MALDI-TOF MS (Matrix Assisted Laser Desorption Ionization—Time of Flight), the dendrogram (Figure 1) based on the protein profiles of the bacterial strains as well as 16S rRNA gene phylogeny showed that six strains from the group *B. cereus* complex, are grouped in three different clusters (Figure 2). Only one strain from each cluster was tested for insecticidal activity in order to avoid redundancy.

### 2.2. Insecticidal Activity Prediction

In the strain *Bti* AM65-52 (used as a control strain), all genes were detected. Most of the genes were absent in the strains we isolated. Only the gene cyt1C was detected in the strain 156 gite of the *B. cereus* complex together with the accessory genes p19 and p20. Strain 125 gite of the *B. cereus* complex showed the presence of only the accessory genes p19 and p20, while in the strain 117 gite, we only detected the accessory gene p19.

### 2.3. Screening for Insecticidal Activity

Of the bacterial strains isolated, 14 showed insecticidal activity. The mortality rises with the concentration. With the concentration 6 mg/L at 72 h post-administration, both for the supernatant and pellet fractions, 11/14 of the strains were active against the *A. albopictus* larvae: a total of 10/14 with the supernatant fractions and 1/14 with the pellet fraction (Table 2).

The most active strain using the supernatant fractions was the 125 gite strain of the *B. cereus* complex with 94% larvae mortality. It was followed by *L. fusiformis* with 85% of mortality and then by the strain 117 gite of the *B. cereus* complex with 80% and *V. pantothenticus* with 79% of larvae mortality. *B. clausii*, *B. licheniformis* 45 gite and *D. propionicifaciens* were active with respectively 75%, 71% and 66% larvae mortality followed by the strains 156 gite of *B. sonorensis* and *B. licheniformis* 143 gite with respectively 56%, 51% and 37%. Only one strain, *A. migulanus,* gave larvae mortality with the pellet fraction, with around 56%.

The larvae mortality rate does not change significantly with the mixture of the pellet and supernatant fractions (X2 = 1.2194 and *p* = 0.2694) (Table 2).

Three strains were not active or had low activity against the mosquito larvae, resulting in less than 20% of mortality: *B. borstelensis* with 19%, *P. thiaminolyticus* with 1% and *B. mojavensis* with 0%.

We performed the comparison of the difference by pairs including the reference strain (Table S1) and found that the difference was significant (critical value of khi^2^ = 23,685, *p*-value ≤ 0.0001) between the insecticidal activity exhibition rates of the supernatant fractions from the different strains at 72 h with 6 mg/L. A difference is considered statistically significant when the *p*-values is < 0.05. The efficacy of the strains was compared with that of the *Bti*-positive control (Table 3). Using the Dunn test, the strains were classified into seven groups according to their efficiencies (Paris, France, https://www.xlstat.com). The group G is the most effective, composed of six strains that had 2X the activity of the positive control “mean ranks = [907 to 1079.5]”, *B. cereus* Complex 3 125 gite, *L. fusiformis*, *B. cereus* Complex1 117 gite, *V. pantothenticus*, *B. clausii* and *B. licheniformis* 45 gite. Meanwhile, the group A is the least effective group and is composed of the lowest and non-effective strains (Table S1) “mean ranks = [374.5 to 517]”.

Ten strains were significantly (*p* < 0.0001–0.011) more active by killing more than 51% of *A. albopictus* larvae compared with the reference strain of *Bti* (33%) (groups C, D, E, F and G), while one strain was non-significantly active (*p* < 0.572), killing 37% of larvae, therefore forming group C with *Bti*AM65-52 (Table 3).

## 3. Discussion

In a recent study investigating the microbiota of four breeding sites in the Amazon basin in Brazil, a high diversity of bacteria was found and all sites were 94% dominated by Proteobacteria and Firmicutes [17]. Most species belonging to these phyla may be entomopathogenic, especially of the *Bacillales* order.

Interesting secondary metabolites were isolated from bacteria and exhibited the highest insecticidal activity, such as phthalic acid from the culture extract of *Photorhabdus temperata* M1021 [18].

We explored the presence of *cyt* and *cry* genes including accessory genes encoding dipteran-specific endotoxins for the insecticidal activity prediction using a set of recently designed primers (Table 4). The commercialized strain *Bti* (strain AM65-52) was confirmed to carry all these genes. Most of these genes are absent in the strains we isolated. Despite the high insecticide activity displayed by all three *B. cereus* complex strains, only one gene (*cyt1C*) was detected in the strain 156 gite of the *B. cereus* complex in addition to the accessory genes *p19* and *p20*, so insecticidal activity is expected in this strain. In the strain gite 125, we detected only the accessory genes *p19* and *p20*, while in the strain gite 117, we detected only the accessory gene *p19*. In this case, insecticidal activity was not expected in these strains and revealed that toxicity can partially be explained by the presence of other endotoxins [16].

The insecticidal activity increased with the higher concentration of bacterial products. A small difference was found between the effect induced by the supernatant only, or the mixture of the supernatant and pellet fractions. The small increase may be induced by active compounds trapped inside the cells and released after the inactivation of the pellet.

VectoBac GR based essentially on *Bti* AM65-52 is the only product to complete the World Health Organization Pesticide Evaluation Scheme (WHOPES). The strain *Bti*AM65-52 was isolated from the product and was then tested to confirm our protocol and to asses our strains’ effectiveness. Its pellet was inactive against *A. albopictus* larvae, while its supernatant fractions at 6 mg/L gives 33% of larvae mortality. We used only 6 mg/L of concentrated sterile culture medium to avoid banal bacterial multiplication in the flasks containing the larvae. Shorter durations and the highest effectiveness must be obtained by the highest concentrations.

Indeed, a large number of studies have been conducted on large collections of *Bacillus* strains to explore their biological activities, including antibacterial, insecticidal and other biological activities [13,14,15,16], for the interests they present such as the antimicrobial substances produced by *B. licheniformis*, *B. pumilus*, *B. circulans*, *B. cereus*, *Brevibacillus laterosporus*, *Paenibacillus polymyxa* and other species [19], or secreted toxins and insecticidal proteins [16]. Several other *Bacillus* spp. exhibited high toxicity against dipterans such as *B. circulans* [20] and *Brevibacillus laterosporus* [21,22].

Three tested strains of the *B. cereus* complex (Figure 2) provided a very high larvae mortality rate. This potent insecticidal activity may be induced by new endotoxins as long as the genes coding for known toxins are not detected in these strains. Several studies exploring *Bacillus* collections reported new strains [16]. Moreover, there can be five or six plasmids in some single *B. thuringiensis* strains, which can encode different toxin proteins [23].

*L. fusiformis* [24] is a *Bacillus*-like bacterium belonging to the family *Bacillaceae* and considered a naturally occurring bacterium, which can be isolated from several environmental samples, such as plants, soil and a few animals [25]. Numerous other interesting *Lysinibacillus* spp. were well-described such as *L. xylanilyticus* [26] and *L. sphaericus* [27]. We tested the supernatant and pellet fraction of *L. fusiformis* that we isolated from the larvae breeding site. The pellet fraction was not active, while the supernatant fraction kills 85% of the *A. albopictus* larvae. Some studies have reported the biological effects of *L. fusiformis,* such as antibacterial activity against resistant microorganisms, and the increasing complexity of biofilms [28,29]. However, in our study, we screened its secondary metabolites for insecticidal activity for the first time.

*V. pantothenticus* is a Gram-positive, spore-forming, aerobic, mesophilic and halotolerant bacterium [30]. This strain has never been explored for potent biological activities in order to be used for strategic intentions. We tested its supernatant and pellet fraction against the *A. albopictus* larvae and found that, interestingly, the supernatant fraction kills 79% of the third and early fourth larvae instars, while the pellet was not active. This strain must be further explored for other biological properties and advanced studies are needed to characterize the active compound against the mosquito larvae.

*B. clausii* is a non-pathogenic, spore-forming, aerobic, Gram-positive bacterium which is found to be beneficial to humans [31]. More precisely, it is effective and safe in the treatment and prevention of acute diarrhea without causing any adverse effects and is actually used as a probiotic [32]. Moreover, some strains were found to release antimicrobial substances active against *Staphylococcus aureus*, *Enterococcus faecium* and *Clostridium difficile* [33]. For the first time in 2015, cyclic lipopeptides produced by *B. clausii* DTM1, exhibited herbicidal activity in addition to insecticidal activity against aphid species *Plutella xylostella* and *Diabrotica balteata* [34]. Further, the strain BS02 was active against pulse beetle and mealybug [35]. We isolated a strain of *B. clausii* from the larvae breeding site and, when tested on the mosquito larvae, its supernatant fraction exhibited a high insecticidal activity with 75% of larva mortality. For the first time, *B. clausii* was shown to be insecticide against mosquito larvae and further studies need be conducted to determine whether the components already identified are responsible for the mosquito larval mortality or if other probably new molecules are the origin. Its insecticidal activity against the larvae of other species of mosquitoes of medical and veterinary interest is to be expected.

Since a long time, *B. licheniformis* strains are known as a source of carbohydrase and protease enzyme preparations used in food processing and form an attractive host for the expression of cloned gene products on an industrial scale [36]. Recently, numerous novel properties were found in some strains, such as the production of antifungal proteins for the biocontrol of plant diseases [37] and their use for crop improvement [38], in addition to the bioremediation of malathion-contaminated soil [39]. We isolated two strains of *B. licheniformis* from the larvae breeding site and when tested on mosquito larvae, their pellets were not active, while their supernatant fractions activities were significantly higher for the strain 45 gite with 71% larvae mortality, in contrary to the strain 139 gite, which killed 37% of the *A. albopictus* larvae. Only few projects studied *B. licheniformis* in insect pest control. In 2003, some strains were transformed to express *B. thuringiensis* δ-endotoxin *cry1Ab* gene [40]. Furthermore, a newly discovered crystalline inclusion, that closely resembles the parasporal crystals of *B. thuringiensis*, suggests that it could be a gene transfer to them from *B. thuringiensis* [41]. The same study suggests that *B. licheniformis* adapts better naturally in phylloplane and that if potential insecticidal properties are discovered from *B. licheniformis*, they will make better candidates for insect biocontrol. To the best of our knowledge, our study is the first to identify the insecticidal properties of *B. licheniformis*’ secreted metabolites. These mosquitocidal compounds must be identified in future studies in order to be used for *A. albopictus*-borne diseases control, but also to be tested on other mosquito species and insect pests.

*D. propionicifaciens* is a Gram-negative, non-motile, non-sporulating bacterium with a convoluted cell surface, which was described for the first time in 2005 [42]. We isolated this strain from the larvae breeding site despite being often found in clinical samples. We found that the supernatant fraction of *D. propionicifaciens* exhibited a significant and good insecticidal activity with 66% larvae mortality, although very few data are available on this bacterium.

*Aneurinibacillus* is a genus uniting aerobic, rod-shaped, endospore-forming bacteria [43]. *A. migulanus* is a soil-borne species which is known for its abilities to control plant disease development through the production of the cyclic peptide gramicidin S and new gramicidin secondary metabolites, increasing its biocontrol activity, and a form a biosurfactant, reducing the surface wetness of plants [44,45,46]. Moreover, it could play a vital role in degrading chemical insecticide profenofos in soil [47]. However, to our knowledge, it has never been studied to identify insecticidal compounds. We tested the secondary metabolites of the *A. migulanus* strain we isolated and found that the pellet fraction exhibited a good larvicidal activity corresponding to either the inclusions inside the bacterium or to the constituents of the cells that we released during the pellet inactivation. In our study, *A. migulanus* is the unique strain that exhibited *A. albopictus* larvae mortality by its pellet faction and not with the supernatant, suggesting that it probably does not secrete secondary metabolites with insecticidal activity. Advanced studies are needed to look for the larvicide compound.

*B. sonorensis* is a rod-shaped, Gram-positive, motile and spore forming bacterium [48]. Very little information is available about this bacterium, although its lipase from a marine strain is used as an additive in detergent formulation [49]. We were interested to test the strain we isolated for insecticidal activity. The pellet fraction is not active against the *A. albopictus* larvae, while the supernatant fraction exhibited a good insecticidal activity, killing off 51%. The properties of this bacterium are not well-known. We have identified for the first time a strain of *B. sonorensis* that produces insecticidal compounds which we need to explore further to characterize them.

Three of the strains we tested did not show insecticidal activity with either the pellet or the supernatant fraction: *B. borstelensis*, *P. thiaminolyticus* and *B. mojavensis*.

*B. borstelensis* is involved in the degradation of the herbicide sulfosulfuron and the biodegradation of polyethylene, useful for future environmental/biotechnological applications. [50,51,52]. In this study, we tested it for the first time for insecticidal activity and it was found to exhibit a low effect against *A. albopictus* larvae.

*P. thiaminolyticus* is a Gram-positive, aerobic bacterium which is related to *Bacilli* and identified as a new cause of human infections [53]. Some *Paenibacillus* species had an insecticidal effect against the larvae of pest insects [53,54]. We isolated a strain of *P. thiaminolyticus* and tested its supernatant and pellet fractions against *A. albopictus* larvae. No insecticidal activity was found against *A. albopictus* larvae.

*B. mojavensis* is known for its antimicrobial properties and plant growth promotion [55,56,57], but it has never been tested before for the production of insecticidal compounds.

In conclusion, we explored the observed natural phenomenon of high mortality in a larvae breeding site. We isolated bacteria strains that exhibit the highest insecticidal activity compared with a commercial bacterial insecticide product (*Bti*) and we concluded that most probably one/several of the isolated bacteria may be responsible for the observed mortality. For most strains, this was the first time they were tested against mosquito larvae. In addition, the insecticidal activity of their separated supernatant and pellet fractions is reported for the first time. Advanced analyses and further studies are needed to characterize the insecticidal compounds and mechanisms of action. It will be of great importance to test them against other important insects of medical, veterinary and agricultural interest in order to use them in the control of biological vectors.

## 4. Materials and Methods

### 4.1. Bacteria Isolation

Twenty (20) natural mosquito breeding sites were explored in May 2019 in the city of Marseille, France, looking for the presence of larvae, pupa or cocoons that would indicate the presence of dead or mal-formed mosquito larvae. A phenomenon of natural mortality was observed in one larva breeding site, where hundreds of dead *Aedes* *albopictus* mosquito larvae (1st and 2nd instars) were observed in a garden that had never been treated with insecticides. This breeding site was located in an empty aquarium with the rest of a degraded peat moss. Other breeding sites were identified in the same garden without associated mortality. The content of the mortality-associated larval breeding site was then recovered, homogenized and, thereafter, ten serial dilutions were made to obtain 1/10. The inoculum (50 μL) was seeded on Columbia agar supplemented with 5% sheep blood (bioMérieux, Marcy l’Etoile, France) and incubated under aerophilic conditions at 37 °C for at least 24 h.

### 4.2. Strains Identification

Bacterial species were directly identified from each bacterial colony using matrix-assisted laser desorption ionization-time mass spectrometry (MALDI-TOF MS) (Bruker Daltonics, Bremen, Germany) [58] following the same protocol as previously described by Seng et al. [59] with a Microflex spectrometer (Bruker Daltonics, Leipzig, Germany). A score of >2 allowed identification at the species level, and a score of <1.7 was considered as insufficient for the identification at the species level. In this case, 16S rRNA gene was amplified and the amplicon was sequenced. Briefly, DNA extraction was performed using EZ1 DNA kits (Qiagen, Courtaboeuf, France), according to the manufacturer’s protocol. Amplification and sequencing were performed as described in the study by Dahmana et al. [60] using 16S universal primers [61]. The obtained electropherograms were assembled and edited using the ChromasPro1.7.7 software (Technelysium Pty Ltd., Tewantin, Australia), and then the sequences obtained were compared with those available in the GenBank database by NCBI BLAST (http://blast.ncbi.nlm.nih.gov/Blast.cgi).

### 4.3. Fractions Preparation

#### 4.3.1. Bacteria Culture

*Bacillus spp.* strains were stored at −80 °C. The culture was carried out on solid medium Columbia agar + 5% sheep blood (bioMérieux, Marcy l’Etoile, France) under aerobic conditions at 37 °C. Confirmation of species identification was made by MALDI-TOF MS. Thereafter, several colonies were transferred in a sterile condition in 1 litter of liquid medium Tryptic soy broth T8907-1KG (Sigma-Aldrich, Saint-Quentin-Fallavier, France), and were incubated for three days at 37 °C and 110 rpm in a shaker incubator under aerobic conditions. A negative culture control (sterile, bacteria-free medium stored behind the other culture flasks) was performed and regularly checked for clouding, which indicates contamination, and a clear broth after 3 days of incubation confirmed the absence of contamination.

The supernatant–pellet separation was carried out by centrifugation at 8000 g for 20 min at 4 °C using A98813 Bottle Assembly, J-Lite PP with a JLA-8.1000 rotor (Beckman Coulter, Villepinte, France). After centrifugation, the supernatant was immediately filtrated at 0.45 μm and then disposed in 75 mL flasks and frozen horizontally at −80 °C and lyophilized the next day. The lyophilizate was stored at −20 °C until further assays.

#### 4.3.2. Releasing Inclusions and Main Cell Components

The pellet was resuspended in PBS and then distributed in 2 mL cryotubes (Bio-One GmbH, Kremsmünster, Austria). Each tube was subjected to 3 freeze–thaw cycles for 5 min using liquid nitrogen and a hybridization incubator heated to 50 °C. The tubes are centrifuged at 13,000 G for 10 s and the contents are transferred into Eppendorf Safe-Lock Tubes, 1.5 mL (Eppendorf Quality™, Montesson, France) and then subjected to 3 sonication cycles at 50 Hz amplitude for 30 s. Subsequently, ultracentrifugation is carried out at 20,000 G for 20 min at 4 °C. Thereafter, the supernatant is recovered and directly filtered at 0.45μm and stored at −20 °C.

#### 4.3.3. Fractions Used in Larvae Assays

Once the larvae were ready (3rd and early 4th instars), the already prepared sterile fractions (pellet and supernatant) of a bacterium were thawed at room temperature and then the Bradford’s protein test was performed. Subsequently, the volume of fraction administered to the larvae was calculated to have two concentrations of 2 and 6 mg/L in the final volume. The supernatant and pellet fractions of each stain were tested separately at 2 and 6 mg/L corresponding to small volumes of fractions administered to the larvae that did not promote common bacterial multiplication in the flask containing the larvae during the tests. We also tested the mix of the two fractions at 6 mg/L each to see if there is a synergetic effect of the secreted compounds and the cell constituents.

We used *Bacillus thuringiensis* subsp. *israelensis*AM65-52 isolated from a commercial granular formulation (VectoBac GR, Valent Bioscience, Libertyville, IL, USA) to check and validate our protocol and to assess our strains’ insecticidal activity.

### 4.4. Plasmid DNA Isolation and Investigation of Bacterial Genes Encoding Toxins

Total plasmid DNA was isolated from the strains belonging to the *B. cereus* complex using a Smart-Pure plasmid kit (Eurogentec, Angers, France).

Plasmid DNA was used for the PCR to screen for the potential presence of genes encoding insecticidal proteins, such as *Cry* and *Cyt* (Table 4). The PCR amplifications were thus performed [62]. We took the *Bti* AM65-52 as a positive control.

### 4.5. Screening for Insecticidal Activity

*Aedes albopictus* colonies were maintained at 27 ± 0.5 °C and 80 ± 5% relative humidity. Adult mosquitoes were maintained on a constant exposure to 10% sucrose presented through cotton balls changed daily. For egg production, adult female mosquitoes were offered defibrinated human blood (French blood agency, Marseille, France) via a Hemotek membrane feeding system. Larvae were fed Tetra-Min fish food in clear water until the pupal stage.

Seventy-five mL flasks were used for the insecticide screening assays. Twenty-five of the 3rd and early 4th larvae instars were pouted in 100 mL of clear water. All the fractions tests were performed on 25 larvae (N = 4), for a total of 100 larvae as recommended by WHO [67]. Immediately after separating larvae in flasks containing 99 mL of clean distilled water, we added the calculated volume. Larvae were maintained at 27 ± 0.5 °C and not fed until the 24th h. Dead larvae were counted at 24, 48 and 72 h. In each test, 100 larvae were used as a negative control that received no fraction, to eventually assess natural mortality. A strain was considered to have a good effect if it caused 20% mortality.

### 4.6. Statistical Analysis

The Epi Info version 7 program (http://www.cdc.gov/epiinfo/index.html), (Addinsoft 2019) and the XLSTAT statistical and data analysis solution (https://www.xlstat.com) were used to compare mortality rates recorded at 72 h after the administration of 6 mg/L of the supernatant fractions. The Kruskal–Wallis test, comparison of k proportions and pairwise comparison were realized. A difference was statistically significant when *p*-values were ≤0.05. The Dunn procedure (Bilateral test) was performed to separate groups of strains according to their efficiencies.

## Figures and Tables

**Table 1:** Isolated strains and their identification.

Phylum	Order	Genus-Species	Strain Name	CSUR Collection Number	MALDI-TOF Score Value
Firmicutes	*Bacillale*	*Bacillus clausii*	33 gite	Q3263	2.224
		*Bacillus licheniformis*	45 gite	Q3264	2.19
			139 gite	Q3262	2.483
		*Bacillus sonorensis*	46 gite	Q3261	2.310
		*Bacillus mojavensis*	133 gite	Q3258	2.402
		*Bacillus cereus* complex	117 gite	Q3259	2.095
		*Bacillus cereus* complex	156 gite	Q3265	2.135
		*Bacillus cereus* complex	125 gite	Q3260	2.063
		*Brevibacillusborstelensis*	12 gite	Q3257	2.493
		*Lysinibacillus fusiformis*	99 gite	Q3255	2.345
		*Virgibacillus pantothenticus*	91 gite	Q3256	2.129
		*Aneurinibacillus migulanus*	126 gite	Q3254	2.474
		*Paenibacillus thiaminolyticus*	136 gite	Q3253	2.035
	*Selenomonadales*	*Dialister propionicifaciens*	172 gite	Q3287	2.432

**Table 2:** Detailed results of insecticidal activity exhibition of the secondary metabolites on 3rd and early 4th instar *A. albopictus* larvae at 72 h post-administration.

Strain	Code	Pellet	Supernatant		Supernatant + Pellet (6 mg/L)
		(6 mg/L) *	2 mg/L	6 mg/L	
*B. cereus* Complex 3	125 gite	0%	88%	94%	84%
*L. fusiformis*	99 gite	0%	47%	85%	76%
*B. cereus*Complex 1	117 gite	0%	65%	80%	88%
*V. pantothenticus*	91 gite	0%	61%	79%	84%
*B. clausii*	33 gite	0%	48%	75%	84%
*B. licheniformis*	45 gite	0%	60%	71%	92%
*D. propionicifaciens*	172 gite	0%	52%	66%	64%
*B. cereus* Complex 2	156 gite	0%	15%	56%	64%
*A. migulanus*	126 gite	56%	0%	5%	52%
*B. sonorensis*	46 gite	0%	24%	51%	56%
*B. licheniformis*	139 gite	0%	16%	37%	40%
*B. borstelensis*	12 gite	0%	15%	19%	16%
*P. thiaminolyticus*	136 gite	0%	0%	01%	00%
*B. mojavensis*	133 gite	0%	0%	0%	0%
*Bti*	AM65-52	0%	15%	33%	34%

*: Both of the concentrations were used. The results reported are those of 6 mg/L.

**Table 3:** The efficacy of the strains is compared with that given by the positive control *Bti*.

Strain	Code	Mortality Rate 6 mg/L	STDEV	Mean Rank	*p*-Value	Decision
*Bti*	AM65-52	33%	Ref	Ref	Ref	Ref
*B. cereus* Complex 3	125 gite	94%	0.239	−457.5	≤0.0001	Pos. S *
*L. fusiformis*	99 gite	85%	0.359	−390	≤0.0001	Pos. S *
*B. cereus* Complex 1	117 gite	80%	0.402	−352.5	≤0.0001	Pos. S *
*V. pantothenticus*	91 gite	79%	0.409	−345	≤0.0001	Pos. S *
*B. clausii*	33 gite	75%	0.435	−315	≤0.0001	Pos. S *
*B. licheniformis*	45 gite	71%	0.456	−285	≤0.0001	Pos. S *
*D. propionicifaciens*	172 gite	66%	0.476	−247.5	≤0.0001	Pos. S *
*B. cereus* Complex 2	156 gite	56%	0.499	−172.5	0.001	Pos. S *
*A. migulanus*	126 gite	56%	0.499	−172.5	0.001	Pos. S *
*B. sonorensis*	46 gite	51%	0.502	−135	0.011	Pos. S *
*B. licheniformis*	139 gite	37%	0.485	−30	0.572	NS ***
*B. borstelensis*	12 gite	19%	0.394	105	0.048	Neg. S **
*P. thiaminolyticus*	136 gite	01%	0.1	240	≤0.0001	Neg. S **
*B. mojavensis*	133 gite	0%	0	247.5	≤0.0001	Neg. S **

* Pos. S: positively significant; ** Neg. S: negatively significant; *** NS: non-significant; STDEV: standard deviation.

**Table 4:** List of primers used in this study to explore the presence of genes encoding endotoxin, accessory proteins and Cyt.

Gene	Primers	Sequences	Amplicon Size	References
*cry4a/4b*	dip2a	GGTGCTTCCTATTCTTTGGC	1290 bp	[63]
	dip1b	ATGGCTTGTTTCGCTACATC		
*cry10*	cry10-1	ATATGAAATATTCAATGCTC	614 bp	[64]
	cry10-2	ATAAATTCAAGTGCCAAGTA		
*cry11*	cry11-1	TTAGAAGATACGCCAGATCAAGC	304 bp	[10]
	cry11-2	CATTTGTACTTGAAGTTGTAATCCC		
*cyt1a*	cyt1A1	GTTGTAAGCTTATGGAAAAT	701 bp	[65]
	cyt1A2	TTAGAAGCTTCCATTAATA		
*cyt1c*	cyt1C1	CAAAATCTACGGGAGCAAGG	1320 bp	[16]
	cyt1C2	GGAAGGATCCCTTTGACTTTT		
*cyt2a*	cyt2A1	AATACATTTCAAGGAGCTA	471 bp	[66]
	cyt2A2	TTTCATTTTAACTTCATATC		
*p19*	p19-1	GCAGGAGGAACATCACCATT	291 bp	[16]
	p19-2	GGATTTGCTGAGCAGGTCAT		
*p20*	p20-1	TGACGAGGAAACAGAGTATACGA	704 bp	[16]
	p20-2	TGAAAGGTTAAACGTTCCGATT		

## Supplementary Materials

The following are available online at https://www.mdpi.com/2076-0817/9/6/486/s1, Table S1: Dunn pairwise test’s results. Comparison of insecticidal activity rates between the different isolated strains and *Bti* (AM65-52).
