# Supplementary material for: Insecticidal Activity of Bacteria from Larvae Breeding Site with Natural Larvae Mortality: Screening of Separated Supernatant and Pellet Fractions
Source: Pathogens. 2020 Jun 18;9(6):486. doi: 10.3390/pathogens9060486 (PMC7350308; doi:10.3390/pathogens9060486)
Supplement: Supplementary file 1 [file pathogens-09-00486-s001.pdf]

		p-value														
		B. borstelensis	***D. propionicifaciens***	***B. clausii***	***B. licheniformis* 45 gite**	***B. licheniformis* 139 gite**	***B. sonorensis***	***L. fusiformis***	***V. pantothenticus***	***B. mojavensis***	***A. migulanus***	***P. thiaminolyticus***	**Complex *cereus* 125 gite**	**Complex *cereus* 117 gite**	**Complex *cereus* 156 gite**	***Bti* AM65-52**
**Mean rank**	***B. borstelensis***		**< 0,0001**	**< 0,0001**	**< 0,0001**	**0,011**	**< 0,0001**	**< 0,0001**	**< 0,0001**	**0,007**	**0,011**	**0,011**	**< 0,0001**	**< 0,0001**	**< 0,0001**	**0,048**
	D. propionicifaciens	**352,500**		**0,203**	**0,480**	**< 0,0001**	**0,034**	**0,007**	**0,066**	**< 0,0001**	**< 0,0001**	**< 0,0001**	**0,048**	**0,157**	**< 0,0001**	**< 0,0001**
	B. clausii	**420,000**	**67,500**		**0,572**	**< 0,0001**	**0,001**	**0,157**	**0,572**	**< 0,0001**	**< 0,0001**	**< 0,0001**	**0,480**	**0,007**	**0,007**	**< 0,0001**
	***B. licheniformis* 45 gite**	**390,000**	**37,500**	**-30,000**		**< 0,0001**	**0,005**	**0,048**	**0,258**	**< 0,0001**	**< 0,0001**	**< 0,0001**	**0,203**	**0,034**	**0,001**	**< 0,0001**
	***B. licheniformis* 139 gite**	**135,000**	**-217,500**	**-285,000**	**-255,000**		**0,048**	**< 0,0001**	**< 0,0001**	**< 0,0001**	**1**	**< 0,0001**	**< 0,0001**	**0,007**	**< 0,0001**	**0,572**
	B. sonorensis	**240,000**	**-112,500**	**-180,000**	**-150,000**	**105,000**		**< 0,0001**	**< 0,0001**	**< 0,0001**	**0,480**	**< 0,0001**	**< 0,0001**	**0,480**	**< 0,0001**	**0,011**
	L. fusiformis	**495,000**	**142,500**	**75,000**	**105,000**	**360,000**	**255,000**		**0,396**	**< 0,0001**	**< 0,0001**	**< 0,0001**	**0,480**	**< 0,0001**	**0,203**	**< 0,0001**
	V. pantothenticus	**450,000**	**97,500**	**30,000**	**60,000**	**315,000**	**210,000**	**-45,000**		**< 0,0001**	**< 0,0001**	**< 0,0001**	**0,888**	**0,001**	**0,034**	**< 0,0001**
	B. mojavensis	**-142,500**	**-495,000**	**-562,500**	**-532,500**	**-277,500**	**-382,500**	**-637,500**	**-592,500**		**< 0,0001**	**0,888**	**< 0,0001**	**< 0,0001**	**< 0,0001**	**< 0,0001**
	A. migulanus	**-105,000**	**-457,500**	**-525,000**	**-495,000**	**-240,000**	**-345,000**	**-600,000**	**-555,000**	**37,500**		**0,572**	**< 0,0001**	**< 0,0001**	**< 0,0001**	**< 0,0001**
	P. thiaminolyticus	**-135,000**	**-487,500**	**-555,000**	**-525,000**	**-270,000**	**-375,000**	**-630,000**	**-585,000**	**7,500**	**-30,000**		**< 0,0001**	**< 0,0001**	**< 0,0001**	**< 0,0001**
	**Complex *cereus* 125 gite**	**457,500**	**105,000**	**37,500**	**67,500**	**322,500**	**217,500**	**-37,500**	**7,500**	**600,000**	**562,500**	**592,500**		**0,001**	**0,048**	**< 0,0001**
	**Complex *cereus* 117 gite**	**277,500**	**-75,000**	**-142,500**	**-112,500**	**142,500**	**37,500**	**-217,500**	**-172,500**	**420,000**	**382,500**	**412,500**	**-180,000**		**< 0,0001**	**0,001**
	**Complex *cereus* 156 gite**	**562,500**	**210,000**	**142,500**	**172,500**	**427,500**	**322,500**	**67,500**	**112,500**	**705,000**	**667,500**	**697,500**	**105,000**	**285,000**		**< 0,0001**
	***Bti* AM65-52**	**105,000**	**-247,500**	**-315,000**	**-285,000**	**-30,000**	**-135,000**	**-390,000**	**-345,000**	**247,500**	**210,000**	**240,000**	**-352,500**	**-172,500**	**-457,500**	

Table S1 : Dunn pairwise test's results. Comparison of insecticidal activity rates between the different isolated strains and *Bti* (AM65-52).
